# Pre-surgical CT-assessment of neurogenic myositis ossificans of the hip and risk factors of recurrence: a series of 101 consecutive patients

**DOI:** 10.1186/s12891-016-1294-2

**Published:** 2016-10-18

**Authors:** Bruno Law-ye, Chloé Hangard, Adrien Felter, Dominique Safa, Philippe Denormandie, François Genet, Robert-Yves Carlier

**Affiliations:** 1 APHP, Neuroradiology Department, Pitié-Salpêtrière Hospital, Paris, France; 2 Pierre and Marie Curie Faculty of Medicine, Sorbonne Universités, Paris, France; 3 APHP, Radiology Department, Neuro-musculoskeletal Pole, Raymond Poincaré Hospital, 92380 Garches, France; 4 Versailles University, Paris-Saclay UMR 1179, End: icap, Saint-Quentin-en-Yvelines, France; 5 APHP, Orthopedic surgery Department, Neuro-orthopedic Unit, Neuro-musculoskeletal Pole, Raymond Poincaré Hospital, 92380 Garches, France; 6 APHP, Rehabilitation Department and Pole, Raymond Poincaré Hospital, 92380 Garches, France; 7 CIC 1429, Raymond Poincaré Hospital, 92380 Garches, France

**Keywords:** Osteoma, Neurogenic myositis ossificans, Paraplegia, Spinal cord injury, Brain trauma

## Abstract

**Background:**

Neurogenic Myositis Ossificans (NMO) is a rare disabling pathology characterized by peri-articular heterotopic ossifications following severe peripheral or central nervous system injuries. It results in ankylosis and vessels or nerves compressions. Our study aimed to describe the pre-operative findings of patients with NMO of the hip using biphasic computerized tomography (CT).

**Methods:**

Between 2006 and 2012, we retrospectively analyzed 101 consecutive patients with hip NMO. We analyzed all CTs and surgical reports following a standardized grid depicting the osteoma and its relations with joint capsule, vessels and nerves and bone mineralization. We studied surgical complications and recurrence during follow-up. Chi2-test and Fischer’s test were performed to compare qualitative values with respectively normal and non-normal distribution. Quantitative values were analyzed with a one factor analysis of variance (ANOVA) test. Agreement between pre-surgical CT and surgical observations was evaluated with Cohen’s kappa test.

**Results:**

Correlation between pre-operative CT and surgical findings was excellent regarding relationships with vessels (0,82) and was good concerning relationships with sciatic nerves (0.62) and with joint capsule (0.68). Close contact or disruption of joint capsule (*p* = 0.005), joint space narrowing (*p* = 0.007) and bone demineralization (*p* < 0.001) were correlated with NMO recurrence.

**Conclusions:**

Biphasic enhanced-CT allows pre-operative assessment of NMO with good correlation to surgical observations and helps prevent surgical complications.

## Background

Neurogenic Myositis Ossificans (NMO), also known as neurogenic paraosteoarthropathy (NPOA), is a rare disabling pathology characterized by appearance of heterotopic peri-articular ossifications following severe peripheral or central nervous system injuries and resulting in ankylosis and vessels or nerves compressions [1]. Its first depiction, by Déjerine and Celier, dates back to 1918, in patients who had suffered from spinal cord injury during First World War. It generally affects large joints, often the hip, and can also affect knees, elbows and shoulders. Its early diagnosis is difficult as the signs (such as local inflammation, peri-articular edema, joint stiffness and induration) may be nonspecific and may mimic infection or veno-occlusive disease. NMO represents a turning point during rehabilitation as it may cause severe ankylosis and nerves or vessels compression. At an early stage, heteropic bone formation can be prevented by non-steroid anti-inflammatory agents. At a late stage, the only effective treatment available is the surgical removal of heterotopic bone formations, with risks of per-procedure fractures, hemorrhages and osteochondral lesions. Therefore an exhaustive pre-surgical planning with enhanced CT must be performed.

## Methods

Between 2006 and 2012, we retrospectively analyzed 101 consecutive cases of patients with CT assessment of hip NMO. The radiological reports were established following a standardized reading grid depicting the osteomas and their relationships with joint capsule, femoral and circumflex vessels and sciatic nerves and mineralization of the femoral head. We analyzed the surgical reports and compared them with pre-surgical CT findings. We collected the follow-up data, especially to assess complications and recurrence of NMO. For each patient, we recorded the age, sex, history of the disease, date of the accident and type of accident (brain trauma, spinal cord trauma, stroke, prolonged resuscitation or other).

### Volumetric CT-scan technique

CT-scans were performed with a 16-section scanner (MX IDT 8000, Philips Medical Systems, Best, the Netherlands) and a double-piston power injector (Medtron, Saarbruecken, Germany). Patients installation was crucial for the success of the exam. It was often difficult because of the deformities and required various methods especially the use of cushions. We placed tourniquets around thighs and calves in order to obtain better opacification of the main vessels, not of superficial vessels. We generally catheterized a vein of the elbow using a 20G cannula and performed a biphasic injection using a high-concentration iodinated solution (iomeprol, Iomeron 400 mg/mL, Bracco Imaging, France) including a first injection of 120 mL at the rate of 1.5 mL/s followed by an injection of 80 mL at the rate of 3 mL/s. CT acquisition was performed with a collimation of 16 × 0.75 mm and was triggered 135 s after the start of injection. Voltage was 140kV and amperage 300 mAs/slice. Rotation time was 0,75 s/rotation. Axial reconstructions were obtained 2 mm in thickness every 1 mm. Multi-planar reconstructions were performed as well as volume rendering 3D images with colour encoding according to tissue densities so as the bone appeared white, veins appeared blue and arteries appeared red.

### CT assessment of NMO

**Fig. 1:**
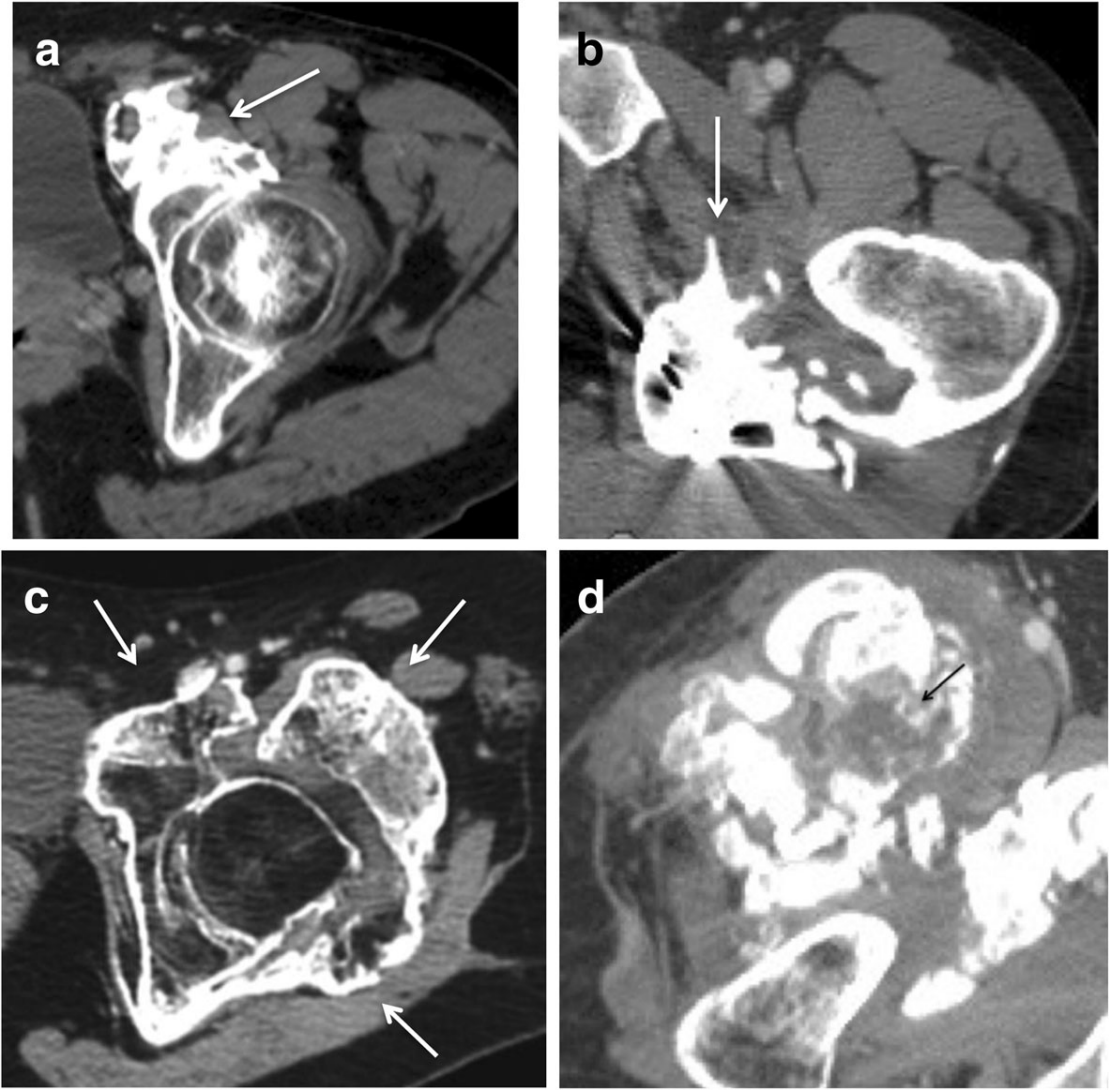
Axial CT images in four different patients. **a** anterior osteoma (*arrow*). **b** posterior osteoma (*arrow*). **c** circumferential osteoma. **d** anterior osteoma in a 31 year-old male patient. Central hypodensity with thin peripheral enhancement corresponding to an immature portion (*black arrow*)

**Fig. 2:**
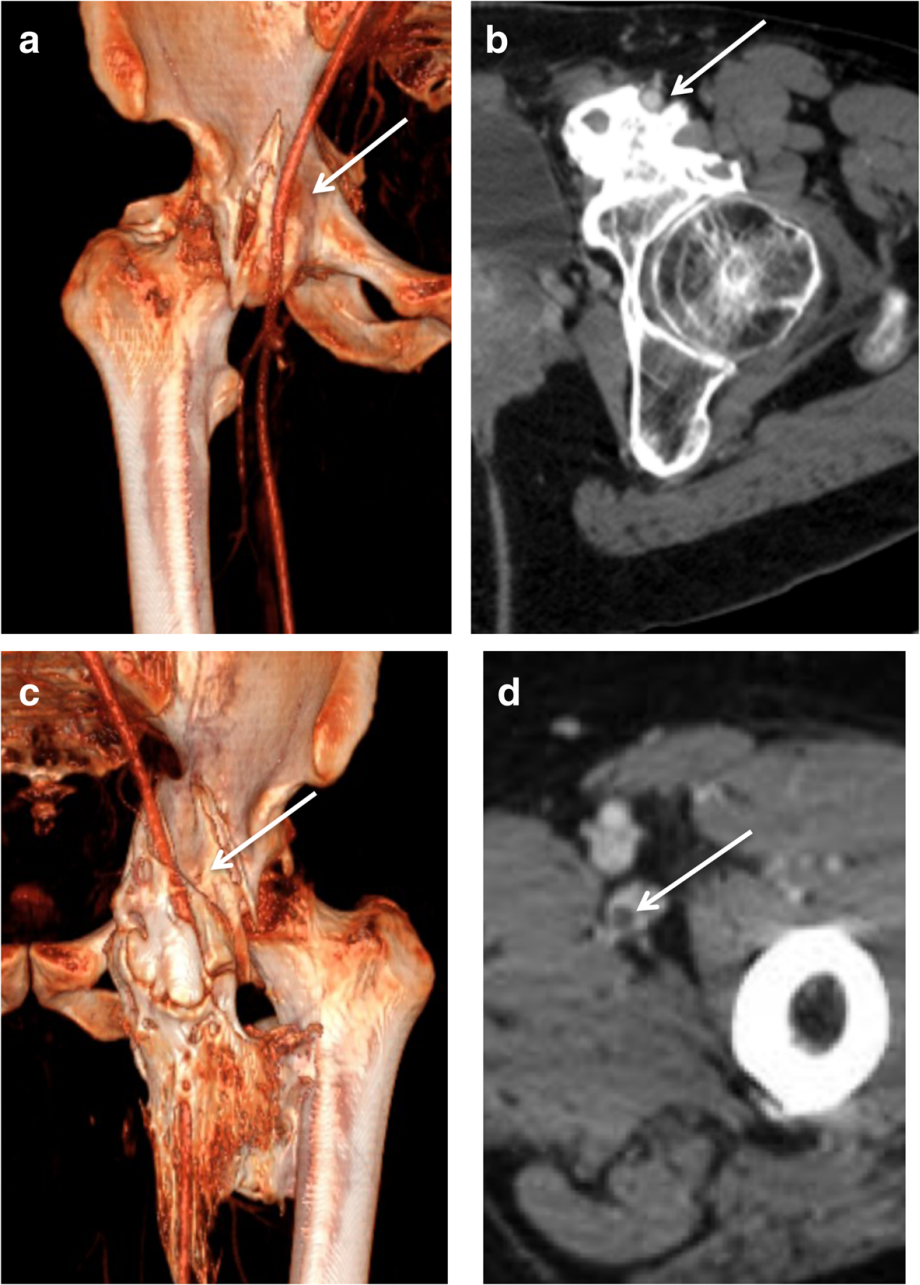
Enhanced-CT in volume rendering reconstruction (**a** and **c**) and axial images (**b** and **d**) illustrating the different types of relationships between osteomas and femoral arteries. **a** displacement of *right* femoral artery with a moderate compression (*arrow*). **b** The osteoma forms a groove surrounding the artery partially, less than 180°(*arrow*). **c** The osteoma forms a complete tunnel around the artery (30 year-old male patient) (*arrow*). **d** Endoluminal defect inside the femoral vein indicating thrombosis (*arrow*)

**Fig. 3:**
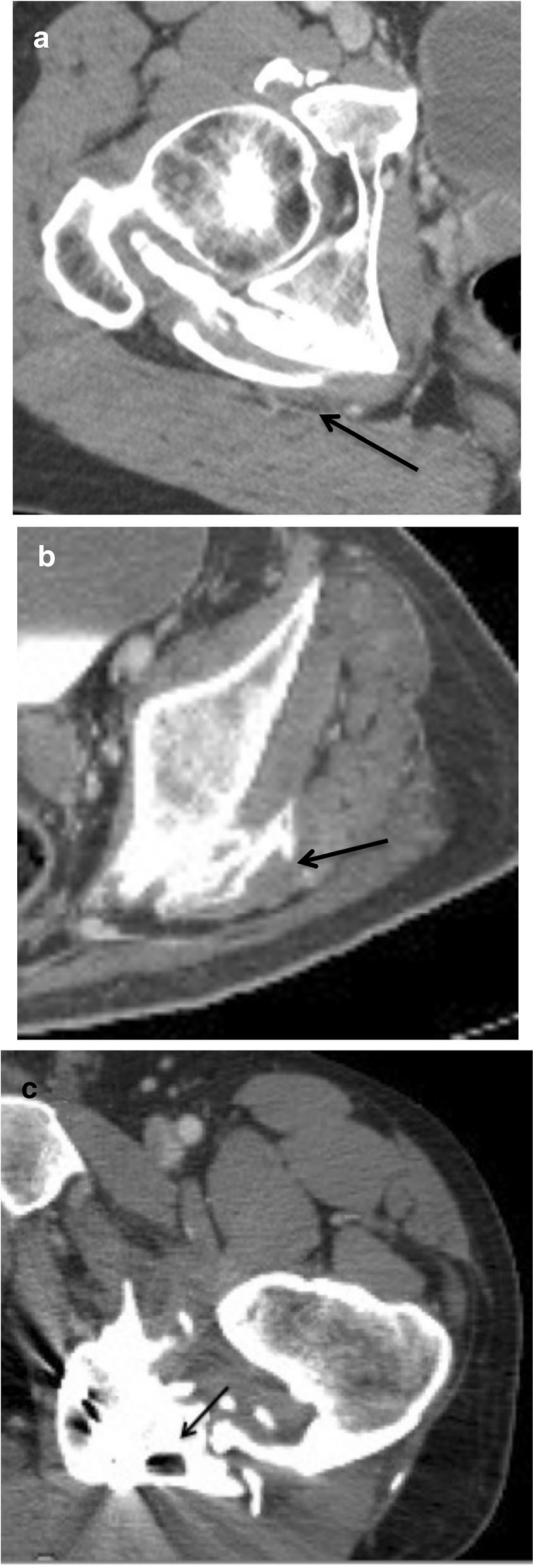
Axial images of a biphasic enhanced CT in three different patients with posterior osteomas with various types of relationships with sciatic nerves. **a** slight contact and compression of the right sciatic nerve by the osteoma (*arrow*). **b** the osteoma forms a groove around the sciatic nerve, surrounding it less than 180° (*arrow*). **c** the osteoma forms a complete bony tunnel around the sciatic nerve (19 year-old male patient) (*arrow*)

**Fig. 4:**
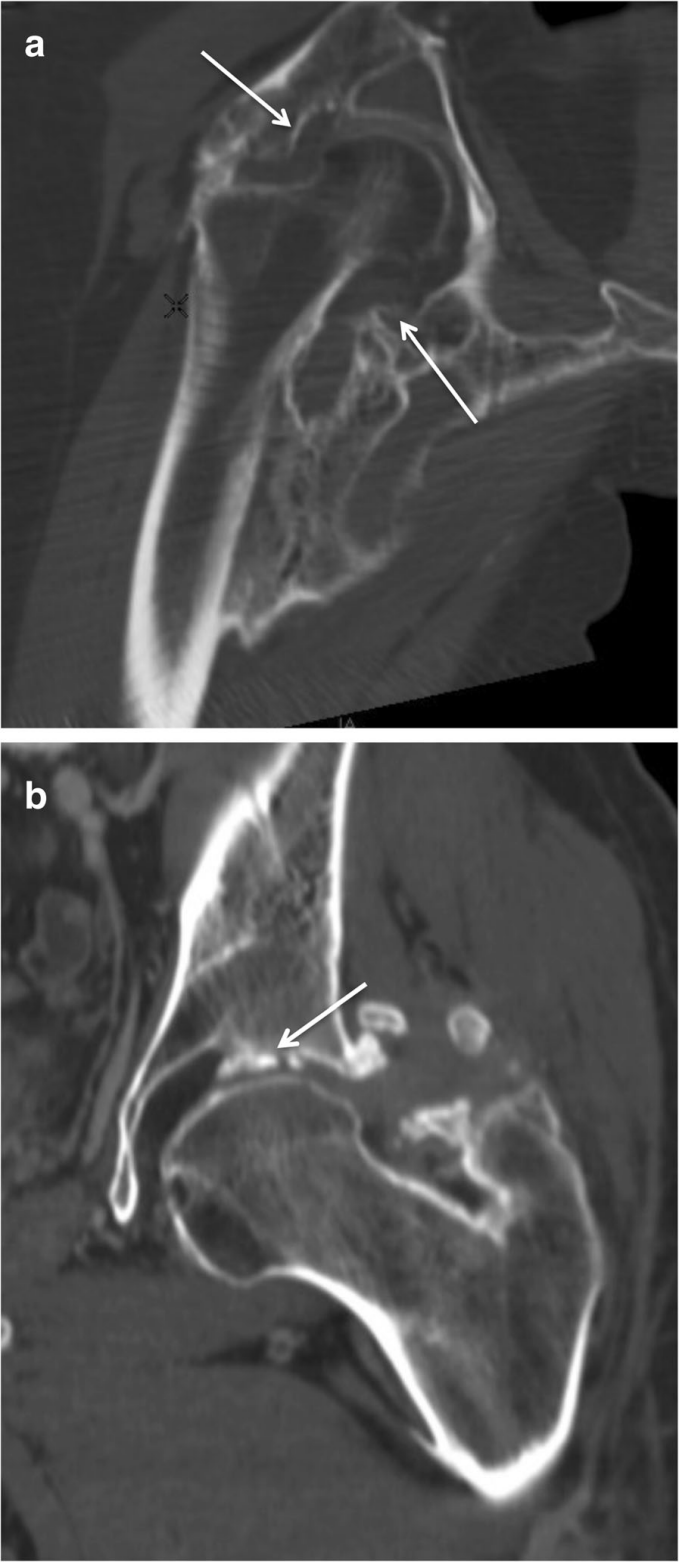
Coronal reconstruction of CT scan: neurogenic osteoarthropathy of the *right* hip (**a**) and *left* hip (**b**, other patient). While the osteoma only touches the joint capsule on image A (*arrows*), we can observe a disruption of joint capsule with an involvement of joint space (27 year-old male patient) (*arrow*)

**Fig. 5:**
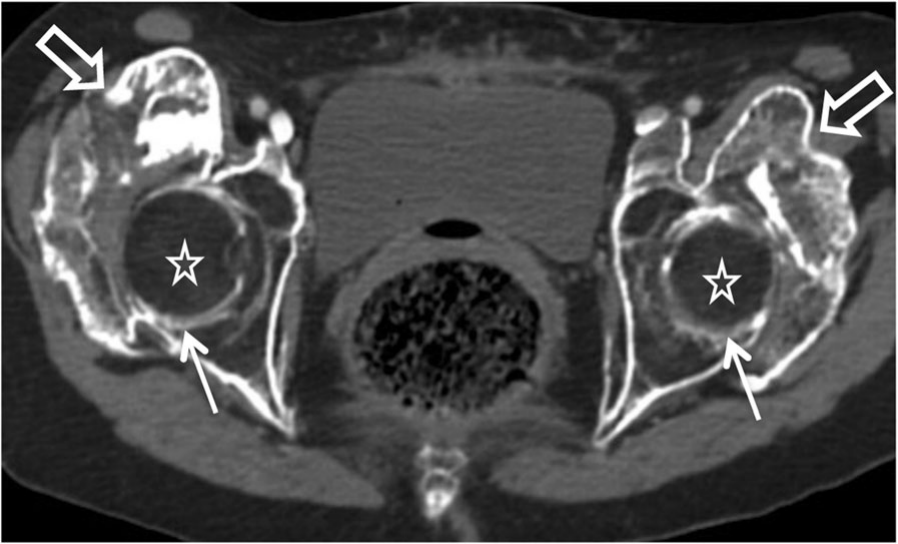
Large bilateral osteomas of the hips on axial image (hollow *arrows*). Advanced demineralization classified M4 (evanescent bone) (*stars*) and collapsed joint space (*arrows*)

The analysis of CT imaging was performed by investigators RC and DS, both of whom have 21 years of experience with this technique. Results are detailed in the Table 1. For each lesion, we determined the implantation base, the location and borders, the presence of maturation zones defined by hypodense non-mineralized tissue (Fig. 1), fragmentation, pseudoarthrosis or intra-lesional neo-joint. We studied the relations to the vessels and classified the either as a slight displacement, a gutter (i.e., a covering of the vessel less than 180° in circumference), a tunnel (engainement of the vessel), a compression or a thrombosis (Fig. 2). Anterior osteomas presented risks for femoral and anterior circumflex vessels while posterior osteomas presented risks for posterior circumflex and gluteal vessels. Apart from hemorrhages, surgical lesions of the circumflex arteries could result in femoral head necrosis. Relationships with nerves were depicted likewise by the following terms: contact, gutter or tunnel. Risks of nervous complications concerned posterior osteomas and sciatic nerves (Fig. 3). Relationships to joint capsules were depicted either as no involvement, touches joint capsule or disrupts joint capsule. Additionally, we reported intra-articular calcifications or ossifications (Fig. 4). Epiphyseal bone mineralization was depicted and classified into four categories: normal (M1), mild demineralization (M2), significant demineralization with a risk of fracture (M3) and severe demineralization (M4), with a replacement of osseous tissue by fatty tissue (Fig. 5). Finally, the joint space was depicted as preserved (S1), narrowed (S2) or ankylosed (S3) [1].

**Table 1:** Depiction of CT and surgical findings

	*N*(%)
CT findings	
Main location	
Anterior	64 (48,5)
Posterior	52 (39,4)
Circumferential	16 (12,1)
Borders	
Sharp	101 (76,5)
Ill-defined	31 (23,5)
Maturation	30 (22,7)
Pseudoarthrosis	56 (42,4)
Monofragmentary	49 (37,1)
Polyfragmentary	83 (62,8)
Relations with vessels	56 (42,4)
Displacement	39 (29,5)
Gutter	6 (4,5)
Tunnel	8 (6)
Thrombosis	3 (2,2)
Relations with nerves	36 (27,2)
Displacement	12 (9)
Gutter	23 (17,4)
Tunnel	3 (2,2)
Relations with joint capsule	85 (64,4)
Contact	61 (46,2)
Disruption	24 (18,2)
Demineralization	
M1	54 (40,9)
M2	51 (38,6)
M3	18 (13,6)
M4	9 (6,8)
Joint space	
Narrowed	33 (25)
Symphysis	17 (12,9)
Total	132 (100)
Surgical findings	
Indications for surgery	
Ankylosis	33 (28,4)
Pain	4 (4,6)
Deformity	20 (23,2)
Recurrence	5 (5,8)
Sciatica	11 (12,8)
Risk of vascular injury	2 (2,3)
Relations with vessels	17(19,7)
Displacement	7 (8,1)
Gutter	4 (4,7)
Tunnel	6 (7)
Relations with nerves	30 (34,9)
Displacement	12 (14)
Gutter	15 (17,4)
Tunnel	3 (3,5)
Contact/joint capsule disruption	16 (18,6)
Surgical complications	7 (8,1)
Haemorrhagic	3 (3,5)
Infection	3 (3,5)
Fracture	0 (0)
Death	1 (1,2)
Recurrence	7 (8,1)
Total	86 (100)

### Surgical assessment and follow-up

Among 101 patients, 80 had surgery within 30 days after the CT scan. We studied all surgical reports and collected the following data: time between accident and surgery, indication for surgery (ankylosis, pain, deformities with flessum, joint limitation, recurrence, sciatica), presence and type of nerves/vessels relationship, joints relations and surgical complications (fractures, hemorrhages). All the patients were seen every six months for follow-up examination to look especially for infections, fractures or recurrence.

### Statistical analysis

Chi2-test was used to comparequalitative variables with normal distribution and Fischer’s test was used for non-normal qualitative values with. Quantitative values were analyzed with a one factor analysis of variance (ANOVA) test. A p value < 0.05 was considered significant. Agreement between pre-surgical CT and surgical observations was evaluated using a Cohen’s kappa test. A coefficient < 0.20 was considered weak agreement, from 0.21 to 0.40 a moderate agreement, from 0.41 to 0.60 a good agreement, from 0.61 to 0.80 a strong agreement and from 0.81 to 1 .00 an excellent agreement.

## Results

### Epidemiology

A hundred and one patients were included in the study. 83 patients were male (82%) and 18 were female (18%). NPOA were bilateral in 31 patients (30%), representing a total of 132 hips. Mean age was 46 years (16-73 years). Etiologies were: brain trauma (*n* = 48 (46.5%)), prolonged resuscitation (*n* = 25 (21.7%)), stroke (*n* = 21 (19.8%)), spinal cord injury (*n* = 8 (7.9%)) and one case of progressive ossifying fibrodysplasia (1%). Two patients (2%) suffered from both spinal cord and brain trauma. Among 132 hips, 86 had surgery (65%). Patients who did not have surgery had either little functional impact or contra-indications (risks of infections and hemorrhages). Main indication for surgery was ankylosis, defined as a single bony bridge, and concerned 33 cases (38.4% of 86). Joint limitation with deformities was the second main indication (20 cases (23.2%)). Five patients were treated for recurrence of NMO. Median time from initial injury to surgery was 21 months (6-285 months). Median time to surgery was 22 months after brain trauma and 20 months after spinal cord injury.

Regarding CT-scans, mean total radiation dose per patient was 555 mGy.cm +/-261 SD and mean effective dose was 8,3 mSv +/- 3,9 SD.

### CT analysis

Osteomas were anteriorly located in 64 hips (48.5% of 132 hips), posterior in 52 hips (39.4%) and circumferential in 16 hips (12.1%). The osteomas had clear margins in 101 cases (76.5% of 132 hips) and irregular infiltrating margins in 31 cases (23.5%). We observed maturation zones in 30 cases. Osteomas were monofragmentary in 49 cases (37.1%) and polyfragmentary in 83 cases (62.8%). Pseudoarthrosis was observed in 56 cases with appearance of a new joint (42.4%).

Contacts with vessels were observed in 56 cases (42.4% of 132 hips), 39 consisted solely of displacements, 6 took the form of gutters and 8 were tunnels. There were 3 cases of common femoral vein thrombosis (Fig. 2d).

All cases of contact with nerves concerned the sciatic nerves. Osteomas were in contact with sciatic nerves in 36 cases (37.2% of 132 hips) including 12 displacements, 23 gutters and 3 tunnels (Fig. 3).

The joint capsule was respected in 48 cases (36.3%). It was touched by the osteomas in 61 cases (46.2%) and disrupted in 24 cases (18.2%).

In most of the cases, bone mineralization was normal (M1) (40.9%). We observed mild demineralization (M2) in 51 patients (38.6%), significant demineralization (M3) in 18 patients (13.6%) and severe demineralization (M4) in 9 patients (6.8%).

Hip joint space was normal in 82 (62%) of 132 hips, narrowed in 33 (25%) of 132 and ankylosed in 17 cases (13%).

### Agreement between CT assessment and surgical observations

Relationships between osteomas and vessels were well depicted in surgery reports. Seven displacements, 4 gutters and 6 tunnels were reported. Agreement with pre-surgical CT was excellent (0.82). Contacts with sciatic nerves were observed by the surgeons in 30 hips (12 displacements, 15 gutters and 3 tunnels). There was a good agreement with pre-surgical CT (0.62). Nine gutters depicted on CT were qualified solely as displacements by the surgeon.

A close contact to joint capsule was reported by the surgeon in 16 hips. Agreement with CT was good (0.68).

### Complications

There were little complications regarding the difficulty of the surgery:

- Two vascular complications: an injury of the circumflex artery and an injury of the femoral vein in a patient with osseous tunnel around the vessels
- One case of hematoma
- Three infections: two cases of abcesses and one arthritis.

Excision of osteoma was incomplete in one patient due to the risk of vessel injury. Patients with M3 and M4 demineralizations presented a high risk of fracture. They had an appropriate management with gentle per- and post-surgical movements. Two patients had a surgical ablation of femoral head and two had osseous grafts on their femoral head. No fracture occurred per- or post-procedure. Mean follow-up after surgery was 10 months. Twelve patients were considered lost for follow-up.

### Recurrence

NMO recurrence was observed in 7 patients.

The statistical analysis found an association between three characteristics and NMO recurrence: osteomas touching or disrupting the joint capsule (*p* = 0.005), joint space narrowing (*p* = 0.007) and demineralization (*p* < 0.001). Though not significant, there was a trend towards recurrence when time to surgery was longer.

## Discussion

NMO are significant complications in patients with paraplegia and other neurological infirmitiesafter brain or spinal cord injury. Their incidence varies from 11% to 22% after brain trauma [2] and from 15% to 20% after spinal cord injury [3, 4]. Symptoms usually occur two to three months after injury [1]. They are usually very disabling and represent a hallmark during rehabilitation. The pathophysiology remains unclear [2, 5, 6]. Venous stasis, associated with bone demineralization may provoke calcium salts precipitation in soft tissues [7]. Some authors postulate that enchondral ossification might be induced by repeated microtrauma due to forced mobilization during nursing and rehabilitation. Preventive treatments exist but are mainly used in post-surgical paraosteoatrhropathies. They include non-steroid anti-inflammatory drugs like indomethacine and radiotherapy [8–11]. An early diagnosis can prevent further ankylosis by beginning anti-inflammatory medication. Once the critical phase is missed, the only effective treatment of ossifications is surgery, in order to facilitate rehabilitation. Risks come both from surgery and from NMO itself (fractures, vascular and nervous complications).

Consequently, the radiologists must establish a detailed and standardized report of all the elements that are pertinent prior to surgery. Enhanced volumetric CT has proven to provide excellent pre-operative assessment of NMO [1].

In our study, the agreement between CT and surgical findings was excellent for vessels and very good regarding relations with joint capsules. Concerning relations with nerves, agreement was average. Indeed in 9 cases in which gutters were described on CT around the sciatic nerves, the surgeons only reported a displacement of the nerves. However, this overestimation was not detrimental to the patients. It indicated to the surgeons to locate and protect the sciatic nerve. Unlike previous studies [1], we observed no fracture in this series. We believe this could be explained by the improvement of pre-operative demineralization assessment and better management of the patients at risk of fracture. Bone densitometry can be artificially increased by heterotopic ossifications [12]. Volumetric quantitative CT should be used to assess bone mineral density [13]. There is no consensus about the optimal timing of surgery. While some authors advise to wait for the NMO to be mature in order to prevent recurrence, we believe this may in turn increase surgical hemorrhages and fractures [12]. Moreover, we observed no relationship between incomplete maturation and recurrence, in agreement with previous studies [13, 14]. In our study, mean time to surgery (21 months) was shorter than the previous study (24 months), probably due to an improved management of the patients and earlier diagnosis. The rate of recurrence was 6%, consistent with previous studies and was lower than the 19.8% rate reported by a meta-analysis [15].

Three main characteristics were associated with recurrence: bone demineralization, involvement of joint capsule and intra-articular lesions. Their late occurrence in the course of NMO evolution seemed to indicate association between late surgery and recurrence. Conversely, 24 of our patients were operated within 12 months with only one recurrence, advocating for an early surgery.

As for other imaging modalities, X-rays are largely insufficient to diagnose NMO. Ultrasonography is an excellent exam for the early diagnosis but appears insufficient for pre-operative assessment. Furthermore, vascular anatomy may be modified by NMO with possible arterio-venous shunts, making Doppler interpretation difficult. MRI could be considered in pre-operative assessment. Diffusion tensor imaging with fiber tracking analysis may provide a better analysis of the sciatic nerve and phase contrast techniques could allow vascular analysis without contrast injection. However, MRI is technically challenging considering the patients’ disabilities. Furthermore, CT remains necessary to study bone structure and osteomas maturity [16] and is an important medium to communicate with surgeons especially with 3D volume rendering reconstructions.

### Limits and further analysis

Our study was retrospective. A future prospective study will certainly allow to confirm our data.

Second, the surgeons’ experience should certainly be taken into account in the good results aside from the imaging work-up. Furthermore, measurement of surgical blood loss would be an interesting parameter to evaluate in further studies.

Finally, relations to tendons should also be studied in future studies as contacts between heterotopic ossifications and adjacent tendons of psoas, gluteus medius and piriformis muscles were reported by the surgeons in 9 cases.

Though difficult to perform in all patients, MRI is undoubtedly the best imaging modality to assess relationships with tendons and tendons﻿' integrity. It might thus be beneficial to conduct MRI following CT whenever the relationships between tendons and osteomas seem problematic for surgery and if MRI is technically possible.

## Conclusion

Enhanced CT with biphasic contrast-injection is a necessary prerequisite for NMO surgery. It provides valuable data to adapt surgical management and prevent complications. The collaboration between radiologists and surgeons and the establishment of a standardized interpretation grid allow a decrease of per- and post-surgical complications.

## Abbreviations

CT: Computed Tomography
MRI: Magnetic resonance imaging
NMO: Neurogenic myositis ossificans
NPOA: Neurogenic paraosteoarthropathy

## References

[1] Carlier RY, Safa DML, Parva P, Mompoint D, Judet T, Denormandie P (2005). Ankylosing neurogenic myositis ossificans of the hip. An enhanced volumetric CT study. J Bone Joint Surg Br.

[2] Pape HC, Marsh S, Morley JR, Krettek C, Giannoudis PV (2004). Current concepts in the development of heterotopic ossification. J Bone Joint Surg Br.

[3] Cassar-Pullicino VN, McClelland M, Badwan DA, McCall IW, Pringle RG, el Masry W (1993). Sonographic diagnosis of heterotopic bone formation in spinal injury patients. Paraplegia.

[4] Singer BR (1993). Heterotopic ossification. Br J Hosp Med.

[5] Ebinger T, Roesch M, Kiefer H, Kinzl L, Schulte M (2000). Influence of etiology in heterotopic bone formation of the hip. J Trauma.

[6] Lal S, Hamilton BB, Heinemann A, Betts HB (1989). Risk factors for heterotopic ossification in spinal cord injury. Arch Phys Med Rehabil.

[7] Major P, Resnick D, Greenway G (1980). Heterotopic ossification in paraplegia: a possible disturbance of the paravertebral venous plexus. Radiology..

[8] Baird EO, Kang QK (2009). Prophylaxis of heterotopic ossification - an updated review. J Orthop Surg.

[9] Schaeffer MA, Sosner J (1995). Heterotopic ossification: treatment of established bone with radiation therapy. Arch Phys Med Rehabil.

[10] Ahrengart L, Lindgren U, Reinholt FP (1988). Comparative study of the effects of radiation, indomethacin, prednisolone, and ethane-1-hydroxy-1,1-diphosphonate (EHDP) in the prevention of ectopic bone formation. Clin Orthop.

[11] Weber EWG, Slappendel R, Durieux ME, Dirksen R, van der Heide H, Spruit M (2003). COX 2 selectivity of non-steroidal anti-inflammatory drugs and perioperative blood loss in hip surgery. A randomized comparison of indomethacin and meloxicam. Eur J Anaesthesiol.

[12] Shehab D, Elgazzar AH, Collier BD (2002). Heterotopic ossification. J Nucl Med Off Publ Soc Nucl Med.

[13] Genêt F, Chehensse C, Jourdan C, Lautridou C, Denormandie P, Schnitzler A (2012). Impact of the operative delay and the degree of neurologic sequelae on recurrence of excised heterotopic ossification in patients with traumatic brain injury. J Head Trauma Rehabil.

[14] Genêt F, Jourdan C, Schnitzler A, Lautridou C, Guillemot D, Judet T (2011). Troublesome heterotopic ossification after central nervous system damage: a survey of 570 surgeries. PloS One.

[15] Chalidis B, Stengel D, Giannoudis PV (2007). Early excision and late excision of heterotopic ossification after traumatic brain injury are equivalent: a systematic review of the literature. J Neurotrauma.

[16] Zagarella A, Impellizzeri E, Maiolino R, Attolini R, Castoldi MC (2013). Pelvic heterotopic ossification: when CT comes to the aid of MR imaging. Insights Imaging..

